# Effects of permissive hypercapnia on intraoperative cerebral oxygenation and early postoperative cognitive function in older patients with non-acute fragile brain function undergoing laparoscopic colorectal surgery: protocol study

**DOI:** 10.1186/s12877-023-04125-4

**Published:** 2023-09-21

**Authors:** Zhichao Li, Youzhuang Zhu, Shangyuan Qin, Xin Gao, Yihan Kang, Si Li, Jun Chai

**Affiliations:** 1 Department of Anesthesiology, Shengjing Hospital of China Medical University, Shenyang, China; 2 Department of Anesthesiology, The Affiliated Hospital of Qingdao University, Qingdao, China; 3 Department of Anesthesiology, Liaoning Cancer Hospital & Institute, Shenyang, China; 4 Department of Anesthesiology, The First Hospital of China Medical University, Shenyang, China

**Keywords:** Permissive hypercapnia, Near-infrared spectrum, Regional cerebral oxygen saturation, Older patients, Nonacute fragile brain function, Randomized controlled trial, Perioperative neurocognitive disorders

## Abstract

**Background:**

Perioperative brain protection in older patients has been the focus of research recently; meanwhile, exploring the relationship between regional cerebral oxygen saturation (rSO_2_) and brain function in the perioperative period has been an emerging and challenging area—the difficulties related to the real-time monitoring of rSO_2_ and the choice of feasible interventions. As an advanced instrument for intraoperative rSO_2_ monitoring, the clinical application of near-infrared spectrum (NIRS) cerebral oxygen monitoring has gradually increased in popularity and is being recognized for its beneficial clinical outcomes in patients undergoing cardiac and noncardiac surgery. In addition, although sufficient evidence to support this hypothesis is still lacking, the effect of permissive hypercapnia (PHC) on rSO_2_ has expanded from basic research to clinical exploration. Therefore, monitoring intraoperative rSO_2_ in older patients with NIRS technology and exploring possible interventions that may change rSO_2_ and even improve postoperative cognitive performance is significant and clinically valuable.

**Methods:**

This study is a single-center randomized controlled trial (RCT). 76 older patients are enrolled as subjects. Patients who meet the screening criteria will be randomly assigned 1:1 to the control and intervention groups. PHC-based mechanical ventilation will be regarded as an intervention. The primary outcome is the absolute change in the percent change in rSO_2_ from baseline to the completion of surgery in the intervention and control groups. Secondary outcomes mainly include observations of intraoperative cerebral oxygenation and metabolism, markers of brain injury, and assessments of patients' cognitive function using scale through postoperative follow-up.

**Discussion:**

The findings of this RCT will reveal the effect of PHC on intraoperative rSO_2_ in older patients with nonacute fragile brain function (NFBF) and the approximate trends over time, and differences in postoperative cognitive function outcomes. We anticipate that the trial results will inform clinical policy decision-makers in clinical practice, enhance the management of intraoperative cerebral oxygen monitoring in older patients with comorbid NFBF, and provide guidance for clinical brain protection and improved postoperative cognitive function outcomes.

**Trial registration:**

ChiCTR, ChiCTR2200062093, Registered 9/15/2022.

## Background

Permissive hypercapnia (PHC), a lung-protective ventilation strategy, can avoid lung injury caused by a high tidal volume and hyperventilation as much as possible and allows a moderate increase in PaCO_2_ and a certain degree of acidemia when maintaining appropriate gas exchange and reducing ventilation pressure is not compatible. Extensive clinical experience has been accumulated on the application of PHC during the treatment of mechanical ventilation-induced acute lung injury and acute respiratory distress syndrome (ARDS) in ICU patients, proving that PHC exerts anti-inflammatory and protective effects on multiple organs [1]. Importantly, as an add-on effect of the lung-protective ventilation strategy, the clinical application of PHC is more correlated than causally related to the purpose of treatment, and the direct lung-protective effect of small tidal volumes and low ventilation pressures is the central focus of the study, with PHC presented more as an incidental outcome and blood gas monitoring indicator. In other words, the current research paradigm mainly attributes the protective effect of these ventilation strategies to the reduction of lung tension alone, whereas hypercapnia is only intended to achieve this goal, and the relative independent clinical significance and acceptability of PHC itself remain widely questioned. However, in recent years, the positive effect of hypercapnia on basic research and perioperative clinical management has received increasing attention.

The traditional view is that a normal or low PaCO_2_ should be maintained during surgery because hypercapnia may lead to tachycardia and elevated blood pressure during the operation, resulting in increased oxygen consumption and a series of adverse perioperative effects. However, an increasing number of preclinical and clinical studies have recently supported the multiorgan-protective effects of hypercapnia, and the protective effect on the brain is a hot topic among these studies [2–6]. PHC can mediate the complex regulation of brain homeostasis in many aspects, such as brain microcirculation and the oxygen supply–demand balance [7–9], brain activity and metabolism [10–12], secretion of excitatory amino acids and neurotransmitters [13], free radical-induced damage [14, 15], the inflammatory response [16, 17], and apoptosis mechanism [6], thus realizing the positive protective effect on brain function. In addition to the extensive basic experimental evidence, several clinical trials have likewise documented the potential for PHC. One study found that the incidence of hypocapnia in patients during general anesthesia reaches 66% and that the postoperative length of hospitalization, morbidity, and mortality is increased in patients with low PETCO_2_ levels during anesthetic management [18]. Moreover, Way and Hill provided evidence that mild hypercapnia (PETCO_2_ values of approximately 40 mmHg or higher) improves the patient prognosis compared to mild hyperventilation and hypocapnia and is associated with a reduced incidence of postoperative complications [19], implying that mild hypercapnia is beneficial and should be accepted as a routine standard of care.

Among these mechanisms of brain protection, maintaining normal brain oxygenation is one of the most critical factors in ensuring normal brain function. However, as a therapeutic ventilation strategy, any beneficial or detrimental effect of PHC on rSO_2_ in patients undergoing major surgery has not been adequately determined. In previous animal model studies, carbon dioxide was an effective vasodilator, particularly in the cerebral vasculature, with a high sensitivity to improve cerebral blood flow (CBF) [20–22]. The definitive neuroprotective mechanisms of mild hypercapnia are still elusive but are presumably associated with increased CBF, enhanced oxygen delivery, and improved cerebral glucose utilization and oxidative metabolism [23, 24]; moreover, mild hypercapnia may activate adenosine triphosphate-sensitive potassium channels to maintain regular neuronal activity during cerebral ischemic events [13]. Furthermore, in a previous study of PHC applied to the management of clinical anesthesia, PHC increased rSO_2_ and reduced the incidence of cerebral oxygen desaturation events (CDE) in patients undergoing general anesthesia [25–27]. This finding further confirms the existence of potential mechanisms by which PHC directly or indirectly affects cerebral oxygenation.

In recent years, NIRS cerebral oximetry has provided a practical method for the continuous noninvasive measurement of rSO_2_. This method has been used in acute and critical care resuscitation, intensive care, and surgical applications with substantial supporting evidence [28–30]. Many studies have also shown that NIRS can be applied during resuscitation and cardiac surgery to effectively monitor and manage hypoxic events in the brain [31–34]. However, although timely intervention is required when certain absolute and relative brain oxygen saturation thresholds are reached [35], these thresholds have not yet been verified, and agreement on the timing and indications for interventions is lacking. A corresponding increase in rSO_2_ has been reported in patients undergoing major surgery with mild hypercapnia, but the exact temporal relationship between intraoperative rSO_2_ and mild hypercapnia is unclear [9].

Notably, Mutch et al. [36] confirmed that the duration and severity of intraoperative hypocapnia are independent risk factors for postoperative cognitive dysfunction (POCD), but researchers have not yet validated whether maintaining hypercapnia during anesthesia is effective at reducing the incidence and severity of POCD and whether it leads to unforeseen adverse events in rigorous clinical trials. POCD is a common neurological complication occurring after anesthesia and surgery and is a recognized clinical phenomenon. In 1955, Bedford described it in the *Lancet* as an adverse effect of anesthesia on the brain of older individuals [37]. POCD is a transient interference of external factors (general anesthesia/surgery, etc.) on the nervous system, often with transient and reversible clinical characteristics. However, some patients may also suffer long-term cognitive impairment. It occurs in patients of all ages but is more common in older patients. The age-related decline of basic cognitive function, neurodegenerative changes, and higher incidence of cardiovascular and cerebrovascular diseases in older patients may be critical factors of age-orientation. Its main clinical manifestations are poor memory, comprehension, and attention after surgery and general anesthesia, which seriously affect the quality of life of patients undergoing surgery, prolong the length of hospital stay and even impose severe mental pressure and an economic burden on families and society [38, 39]. In addition, in recent years, many relevant studies have emerged describing the effect of rSO_2_ on postoperative cognitive outcomes, but the results of these studies are highly controversial.

On the one hand, many studies show that rSO_2_ is closely related to postoperative cognitive impairment and can even be used as an essential predictor of POCD [40–42]; on the other hand, the recent mainstream opinion based on clinical studies and meta-analyses seems to favor the lack of a significant correlation between intraoperative rSO_2_ and POCD [43–45]. However, patients with underlying brain diseases tend to have a worse basic cognitive function status and ability to self-regulate regional CBF and microenvironment homeostasis. Based on this premise, some issues must be reexamined: is the effect of rSO_2_ regulation on postoperative cognitive outcomes more clinically significant? Is the regulation of rSO_2_ one of the primary mechanisms by which PHC protects the brain protection? Is there a potential chain for the following relationship:* "PHC → improves cerebral oxygenation function → improves postoperative cognition"*? The answers to all these questions are inconclusive; thus, a clinical inquiry is vital for practice. Therefore, in selecting trial subjects, NFBF was considered the basic inclusion criterion according to the "*Guidelines for the management of perioperative anesthesia in older patients in China*" [46]. Fragile brain function without definite attack and corresponding clinical symptoms within nearly three months are defined as "non-acute fragile brain function" [*Cerebral Hemorrhage, Cerebral Infarction, Transient Ischemic Attack (TIA), Cerebrovascular Stenosis, Parkinson's Disease (PD), Long-term Headache and Dizziness*]. The qualification of "fragile brain function" and "nonacute" not only satisfies the screening of specific populations with more experimental significance but also ensures the safety and ethical guidelines of the trial as much as possible.

In addition, PETCO_2_ is closely related to PaCO_2_. Continuous noninvasive end-expiratory carbon dioxide monitoring has been used as a reliable indicator to evaluate PaCO_2_ in perioperative mechanically ventilated patients. During mechanical ventilation during general anesthesia, a high PETCO_2_ can be achieved by adjusting the respiratory rate or specified tidal volume, which makes permissible hypercapnia feasible and easy to implement in the management of general anesthesia.

In addition, laparoscopic surgery has become very convenient for older patients with colorectal cancer in recent years. However, CO_2_ pneumoperitoneum during laparoscopic surgery may affect the nervous system of patients and lead to postoperative cognitive dysfunction. In a prospective clinical study of 22 adult women, Liu et al. found that laparoscopic surgery (pneumoperitoneum pressure of 15 mmHg) significantly reduced the Mini-mental State Examination (MMSE) scores and increased the serum S-100β protein level at 1, 6, 12, 24, and 72 h after surgery compared with traditional open surgery [47]. Two potential explanations have been proposed: hypercapnia caused by CO_2_ pneumoperitoneum and the adverse effect of pneumoperitoneum pressure. Admittedly, some conventional views suggest that hypercapnia negatively affects postoperative cognitive outcomes. The theoretical basis of brain edema, high intracranial pressure, central nervous system ischemia, and hypoxia due to the subsequent dilatation of the cerebrovasculature caused by hypercapnia of the CO_2_ pneumoperitoneum seems to provide possible support for this hypothesis. However, the specified pathophysiological mechanism has mainly been explored using basic research, and we must be clear that the situation may be different when the scale of hypercapnia is further refined to permissive hypercapnia. By adjusting the ventilation strategy, the patient is eased into a treatment-acceptable acidic environment, and relatively stable conditions are maintained during the operation by PETCO_2_ and intermittent blood gas measurements. The effect on the patient's body differs substantially from animal models or cellular and molecular models with a rapid/highly acidic environment. This hypothesis is strongly supported by several previous preclinical and clinical studies on the hazards of hypercapnia and the beneficial outcomes of permissive hypercapnia [5, 6, 48–50].

Moreover, pneumoperitoneum pressure is another factor that must not be ignored, and the lower the pneumoperitoneum pressure is, the less of an effect on the body will be observed. Studies have shown that pneumoperitoneum pressure aggravates neuroinflammation and cognitive impairment induced by surgery in aged mice [51]. However, laparoscopic surgery was selected as the surgical method in the present study, indicating that laparoscopic pneumoperitoneum factors were set as control variables, and the independent effects on cerebral oxygen and postoperative cognitive function were unable to be further explored. However, the selection of basic research methods, based on their wide use and practical significance, will undoubtedly further amplify the external effects of the final research conclusions and further improve the ethical practicality.

In summary, when the three key factors of "older patients," "fragile brain function," and "CO_2_ pneumoperitoneum and laparoscopic surgery" are linked together, the protection of the perioperative cognitive function of patients must receive increasing attention. Notably, the change and adjustment of CO_2_ pneumoperitoneum pressure are extremely limited due to the essential requirement of a clear operative field for delicate surgical operations. However, it seems to be a pioneering prospect from the perspective of hypercapnia. First, although permissive hypercapnia was the intervention used in our study, in combination with clinical practice, mild hypercapnia itself is difficult to avoid in major laparoscopic surgery, and extreme ventilation parameter settings (high respiratory rate/high tidal volume) are often required to force normal conditions, which may exert adverse effects. Therefore, our intervention is different from that used in most other RCTs. We evaluated the advantages and disadvantages of both in terms of justifying their existence and acceptability in terms of medical factors, combined with the adverse effects (lung tissue traction injury, airway pressure damage, hyperventilation, respiratory muscle fatigue, tachycardia, etc.) of the forced manual setting of ventilation parameters to achieve a normal blood pH. Previous preclinical and clinical studies of the effects of hypercapnia on the central nervous system and postoperative cognitive function have largely produced negative results; however, further explorations are needed to determine whether this problem can be further improved when hypercapnia is converted to a more precise state of permissive hypercapnia.

Therefore, we will conduct a prospective, single-blind RCT. Laparoscopic colorectal surgery is used as the surgical method and older patients with NFBF are defined according to the "*Guidelines for the management of perioperative anesthesia in older patients in China*" [46], which are the main inclusion criteria used to explore our established hypothesis that the PHC group (H) will exhibit improved brain oxygenation and increased rSO_2_ compared with the target normal group (N) (Table 1). In addition, as a secondary objective, we will evaluate whether PHC affects postoperative cognitive outcomes and further verify the existence of the potential mechanism of "*PHC → improves cerebral oxygenation → improves postoperative cognition*."

**Table 1:** Definition and classification of NFBF

Classification of NFBF	Definition of NFBF
Cerebral haemorrhage	Non-traumatic haemorrhage is caused by rupture of blood vessels in the brain parenchyma, accounting for 20–30% of all strokes
Cerebral infarction	Also known as *cerebral ischemic stroke*, it is an ischemic necrosis or softening of limited brain tissue caused by ischemia and hypoxia due to impaired blood supply to the brain (*Cerebral thrombosis, lacunar infarction and cerebral embolism*)
Transient ischemic attack (TIA)	A transient, limited cerebral deficit or retinal dysfunction caused by a cerebrovascular lesion, with clinical symptoms generally lasting 10–20 min, mostly resolving within 1 h and up to 24 h, with no residual neurological deficit symptoms and no responsible lesions on structural imaging (CT, MRI)
Craniocerebral vascular stenosis	Several factors cause the narrowing of blood vessels in the brain, reducing the amount of blood flow and oxygen supply, and causing localized ischemia and hypoxia in brain tissue, resulting in a series of clinical symptoms (*congenital cerebral stenosis/spastic cerebral stenosis*)
Alzheimer's disease (AD)^a^ ***(exclusion)***	A neurodegenerative disease whose various pathophysiological aspects are still under investigation, characterized by a progressive decline in cognitive function and specific types of neuronal and synaptic loss, the most recognized pathological events are amyloid plaques and neurofibrillary tangles
Parkinson's disease (PD)	A common and complex neurological disorder whose core is a neurodegenerative disease with early prominent death of dopaminergic neurons in the substantia nigra pars compacta (SNpc)
Chronic headache/ Chronic dizziness	Information was obtained from all inquiries during the preoperative visit and hospitalization

Fragile brain function without definite attack and corresponding clinical symptoms within nearly three months are defined as "non-acute fragile brain function"; AD^a^ is often accompanied by severe preoperative cognitive dysfunction, which does not meet the inclusion criteria

## Methods

### Study design, setting and participants

**Fig. 1:**
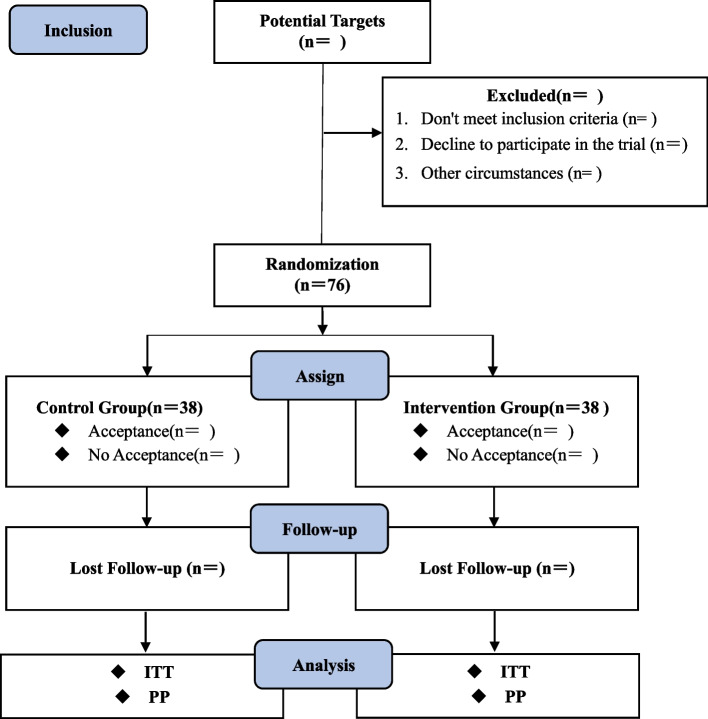
Flowchart of the study

Between October 2022 and July 2023, we plan to conduct an RCT at Shengjing Hospital of China Medical University (Shenyang, China), a university teaching, higher education, large general hospital. The trial period is expected to be 10 months, during which patient recruitment, intervention implementation, and postoperative follow-up will be conducted in an orderly manner according to the actual situation. The final results are expected to be reported in October 2023. Participants will be screened from the patients proposed for elective laparoscopic colorectal surgery in the gastrointestinal surgery department of the hospital. Patients who are eligible for elective major surgery are identified after a preoperative evaluation and receipt of written informed consent before the patient's surgery date. Patients who meet the screening criteria will be randomly assigned 1:1 to the control group (Conventional ventilation group parameters: Tidal volume10 ~ 12 mL/kg, Respiratory rate 14 ~ 16 bpm, Suction/Respiration ratio 1:2, PaCO_2_ 35 ~ 45 mmHg, PEEP 0 cmH_2_O, FiO_2_ 40%) and the intervention group (Permissive hypercapnia group parameters: Tidal volume 6 ~ 8 mL/kg, Respiratory rate 12 ~ 14 bpm, Suction/Respiration ratio 1:2, PaCO_2_ 45 ~ 55 mmHg, pH > . 7.2, PEEP 0 cmH_2_O, FiO_2_ 40%), and 38 patients will be included in each group. Baseline preoperative rSO_2_ values will be recorded, and real-time intraoperative rSO_2_ monitoring will be performed by investigators who are not providing the clinical intervention. In addition, patients will be followed up postoperatively with MMSE scale to assess early postoperative cognitive function. The patients with postoperative MMSE scores ≤ 23 and a decrease of more than 2 points from the baseline value were defined as delayed neurocognitive recovery (DNR). It should be noted that the follow-up personnel have received standardized training in advance, to ensure that the survey scale can be used scientifically and correctly (Fig. 1).

### Protocol amendment

The clinical registration of this trial was 9/15/2022, but in the follow-up protocol design, we identified several problems that needed further refinement and modification. The modified protocol will be more consistent with clinical practice, and the clinical feasibility and significance will be further expanded. In addition, we have completed the amendments of the information in the clinical trial registration platform. The amendments are described below:①In the original registration, we defined the four intraoperative time points for special attention as T_0_-T_3_ and did not specify the method to refer to the baseline rSO_2_ time point. The preoperative baseline rSO_2_ measurement time point is now defined as T_0_, and the four intraoperative time points are changed to T_1_-T_4_ to more clearly and more intuitively define the reference measurement;②In the inclusion criteria listed in the clinical registry, we added "massive intraoperative hemorrhage; intraoperative severe subcutaneous emphysema; operative time < 2.5 h, or > 4 h" as exclusion criteria, which should be an intraoperative contingency to discontinue the trial rather than preoperative screening criteria. In addition, we must clarify several discontinuation conditions in this trial: massive intraoperative hemorrhage; intraoperative severe subcutaneous emphysema; operative time < 2.5 h or > 4 h; CDE; Intraoperative change to open operation; Other events that could threaten the patient's safety;③Regarding the sample size, our calculation method remains unchanged, but the clinically meaningful threshold value of the calculation has been changed from the previous "15%" to a more conservative "10%" and the final sample size was increased from 48 to 76 cases. The specific reasons will be described in the sample size calculation;④Through the pilot trial, we found that the use of postoperative cognitive function assessment methods must be combined with the clinical reality, and the application of the Montreal Cognitive Assessment (MoCA) is challenging. Although the sensitivity of MMSE in detecting mild cognitive impairment is not good enough, it has high clinical practicability. As a secondary outcome measure, we can accept the limitations of the MMSE. Therefore, we will finally choose the MMSE scale as the tool for postoperative cognitive function assessment;⑤The previous description of "Absolute Difference" in the outcome of the main study is not appropriate. We will redefine it as "Absolute Change", that is, the actual increase or decrease between the observed value and the baseline value. The replacement of this term will not affect the rest of the experimental protocol.

### Inclusion and exclusion criteria for the trial

#### Inclusion criteria

- ASA status I-III;
- Age ≥ 65 years;
- BMI 18–28 kg/m^2^;
- Preoperative MMSE score ≥ 23 points;
- Conscious and able to think independently;
- Planned laparoscopic colorectal cancer surgery, no contraindication to surgery;
- Nonacute fragile brain function (*History of Cerebral Hemorrhage, Cerebral Infarction, TIA, Cerebrovascular Stenosis, PD, Long-term Headache and Dizziness, and no attack or corresponding clinical symptoms within three months*);
- Consent to participate in the trial.

#### Exclusion criteria

- Patients with intracranial hypertension (*hydrocephalus, brain tumors, brain-occupying lesions, *etc*.*);
- The forehead has a history of surgery, trauma, lesions, and infection;
- Patients with a history of severe depression, schizophrenia, and other psychoneurological disorders or who have taken antipsychotic or antidepressant medications;
- Patients with intense visual and hearing impairments who are unable to communicate appropriately;
- Patients with a combination of severe underlying diseases other than hypertension, diabetes mellitus, COPD, and underlying conditions related to fragile brain function, as defined in the inclusion criteria (*e.g., acute coronary syndrome, severe cardiac arrhythmias, patients with bitter heart, liver, or renal failure, *etc*.*);
- Patients with preoperative moderate to severe anemia (*Hb* < *90 g/L*);
- Patients with a preoperative arterial partial pressure of oxygen < 70 mmHg;
- Patients undergoing an emergency operation or being converted to an open process due to unforeseen circumstances;
- Patients already involved in other clinical studies may affect the results of this study.

### Randomization and blinding

Random numbers generated by SPSS 25.0 software will be placed in sealed opaque envelopes for patient selection. Patients will be divided into Groups H and N based on random numbers at a 1:1 ratio, and they will not be aware of the grouping. The randomization process will be completed by a study administrator who is not involved in this study. Due to the operational uniqueness of PHC, only patients, data collectors, postoperative followers, and technicians testing blood indicators could be blinded, while clinical interventionists could not. Patients who meet the screening criteria will immediately be randomly assigned to the intervention and control groups. PETCO_2_ will be set accordingly to achieve the desired intervention, but the anesthetist will not have a set rSO_2_ target. Data for all trial outcomes will be collected by an independent researcher unaware of the treatment allocation. After data analysis, the sequences will be decoded. Notably, the anesthetist performing the intervention will not be involved in assessing postoperative cognitive function, and in principle, all study participants should be blinded except for those who are implementing the intervention.

### Outcomes and data collection

#### Primary endpoint

The absolute change in the percent change in rSO_2_ from baseline (T_0_) to completion of surgery (T_4_) in groups H and N.

#### Secondary endpoints

- Intraoperative T_1_, T_2_, T_3_, and T_4_ cerebral oxygen metabolism-related indexes (*rSO*_*2*_*, Da-jvO*_*2*_*ml/L, CERO*_*2*_*%*);
- Incidence of CDE (≥ *20% reduction in cerebral oxygen saturation relative to baseline or absolute rSO*_*2*_ ≤ *55%*);
- MMSE scores 1d before and 1, 3, and 7d after surgery and the incidence of DNR;
- 1d preoperatively (baseline) and 1d postoperatively: serum neuron-specific enolase (NSE) and interleukin-6 (IL-6) levels;
- Time to awaken from anesthesia;
- Postoperative admission rate to ICU;
- The all-cause mortality rate at 30 days postoperatively (*follow-up*);
- Pulmonary complications after surgery (*bronchospasm, atelectasis, exacerbation of chronic lung disease, infection, prolonged mechanical ventilation, and respiratory failure*);
- Duration of the hospital stay;
- Presence of postoperative adverse events (*nausea, vomiting, hypotension, bradycardia, and excessive sedation*): ①A 20% reduction in systolic blood pressure relative to baseline or MAP < 65 mmHg is defined as a perioperative hypotensive event; ②Heart rate < 50 beats/min is defined as bradycardia; ③Level of sedation assessed using the Ramsay scale (*Level 1 awake*: the patient is anxious, restless or irritable; *Level 2 awake*: the patient is cooperative, well-oriented or quiet; *Level 3 awake*: the patient responds only to commands; *Level 4 sleep*: the patient is responsive to tapping between the eyebrows or strong vocal stimuli; *Level 5 sleep*: the patient is unresponsive to tapping between the eyebrows or strong verbal stimuli; *Level 6 sleep*: the patient does not respond to tapping between the eyebrows or strong verbal stimuli), where a Ramsay level ≥ 3 is defined as excessive sedation.

(**T**_**0**_: Immediately after entry, before any treatment **T**_**1**_: 15 min after tracheal intubation **T**_**2**_: 60 min after pneumoperitoneum **T**_**3**_: 120 min after pneumoperitoneum **T**_**4**_: suture completion, end of surgery).

### Measurement of rSO_2_

#### Necessity

The brain is the most metabolically active organ in the body and one of the main sites of action of anesthetic drugs, and the risk of internal homeostatic imbalance and postoperative dysfunction exists after surgery. The brain, the main organ of the central nervous system in our body, is complex and fragile. Although it accounts for only 2–3% of the total body weight, it consumes 15–20% of the standard cardiac output and 20–25% of the whole-body oxygen content. The brain is a highly metabolic, high-oxygen-consuming organ, increasing its sensitivity to hypoxia. The brain is susceptible to various adverse prognostic reactions, including semicoma, coma, sudden illness, memory loss, and intellectual disability. However, most cerebral hypoxic events are difficult to monitor effectively with currently available equipment, and thus the damage to the brain is often long-established by the time the hypoxic event is detected.

Furthermore, as a common consensus, the available research evidence supports that cerebral ischemia and hypoxia may be direct causes of neurocognitive impairment and may play essential roles in the pathophysiological mechanisms of POCD, AD, and neurodegenerative diseases. For example, hypoxia induces the activation of various factors, reduces the cerebrovascular reserve, and exacerbates excitotoxicity and neuroinflammation [52, 53]. The *“2020 American Society for Enhanced Recovery and Perioperative Quality Initiative”* issued a joint consensus statement on the use of perioperative cerebral oxygen monitoring techniques [54] summarizing relevant studies on preoperative baseline rSO_2_ values and postoperative outcomes, and suggested a correlation between low baseline rSO_2_ values and adverse outcomes, such as postoperative neurocognitive impairment, increased 30-day all-cause mortality, and prolonged hospital discharge. The importance and necessity of perioperative cerebral oxygen saturation monitoring are further recommended.

#### Equipment

**Fig. 2:**
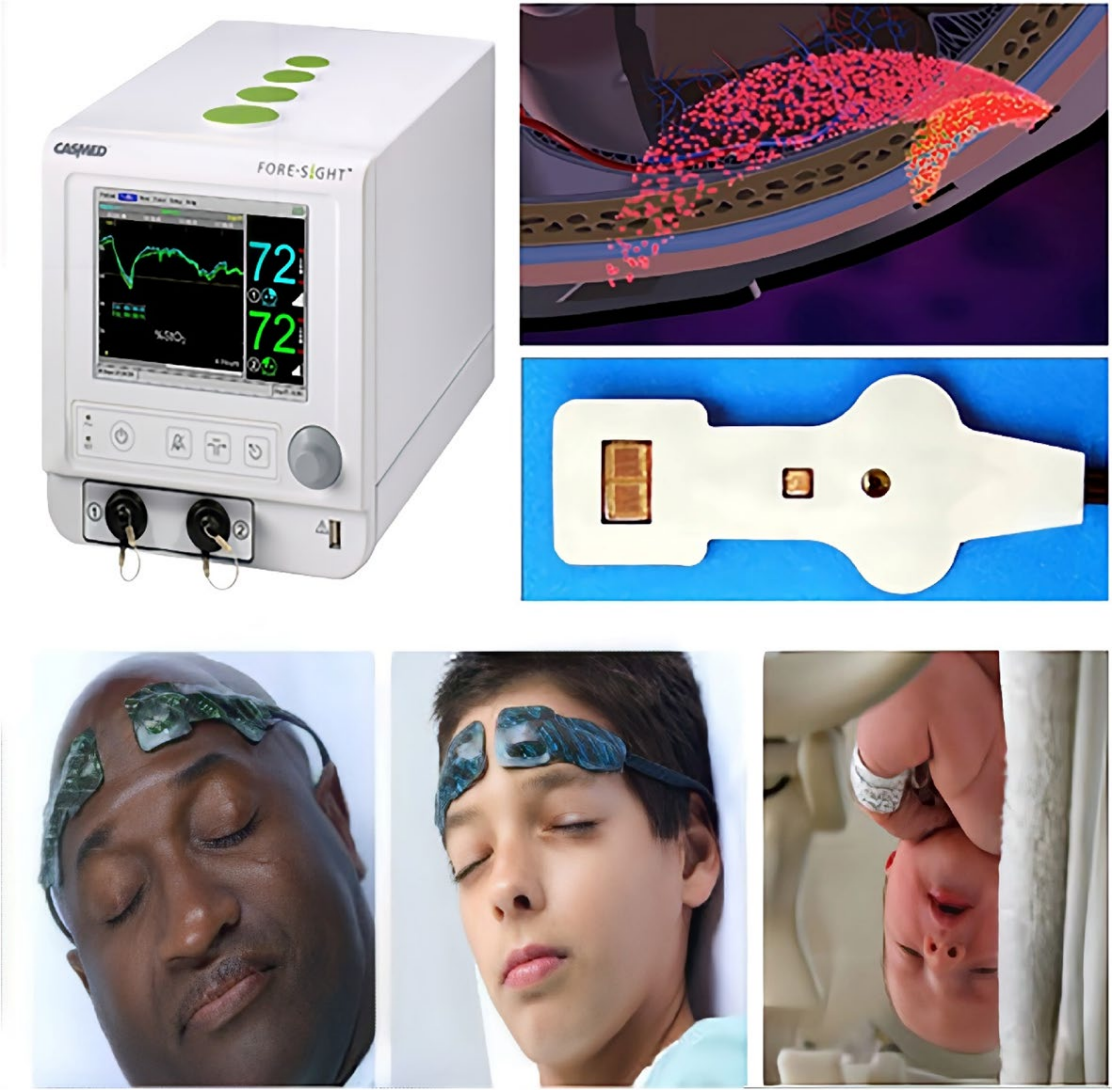
FORE-SIGHT cerebral oxygen monitor and sensors (The copyright of Fig. 2 belongs to *Beijing Gloryway Medical Co., Ltd*, and we have obtained the associated permissions)

The determination of rSO_2_ using NIRS to estimate the oxygen saturation of hemoglobin in brain tissue was first developed in the late 1970s [55] and became commercially available with the introduction of the NIRO-1000 by Hamamatsu Corporation (Iwata, Shizuoka, Japan) in 1989 [56]. With additional clinical research evidence in recent years, the clinical relevance and feasibility of using cerebral oximetry to detect adverse perioperative clinical events and guide patient care in cardiac and noncardiac surgical settings have been initially confirmed [57, 58]. However, NIRS devices from multiple manufacturers differ in wavelength, wavelength number, sensor configuration, and proprietary integration algorithms used to determine brain saturation. Differences between devices make their final measured values not directly interchangeable in equal amounts. The FORE-SIGHT Oximeter (Fig. 2) will be used in this study. Thus, we will briefly explain the corresponding principles of the function and operating methods below.

### Function principles

The FORE-SIGHT measurement method has essential features such as noninvasiveness, continuity (data updated once every two seconds), and the construction of rSO_2_ trend maps (comparison of differences between the left and right hemispheres of the brain) on both sides of the brain. The sensor is available in three sizes, large, medium, and small (Fig. 2), it is adapted to adults, children, and newborns, and the appropriate sensor can be selected for each group relative to the patient's weight. The sensor detects the difference in the absorption of oxyhemoglobin and deoxyhemoglobin using a laser that emits four bands of near-infrared light that penetrate the scalp, skull, and brain tissue, thus measuring the mixed arterial-venous oxygen saturation of the brain tissue. Hemoglobin is a known carrier with oxidation and de-oxidation characteristics, and the degree of oxygen saturation of brain tissue depends on the proportion of oxyhemoglobin in total hemoglobin. The NIRS technique uses laser wavelengths to estimate the ratio of oxyhemoglobin to total hemoglobin by absorbing the fully and partially oxidized hemoglobin molecules differently. The specific algorithm is extremely complex and we will not go into details here (water and hemoglobin are different in human tissue absorption spectra, hemoglobin can be detected at 650 nm—900 nm) (Fig. 2).

### Operating method

In this study, we will use a FORE-SIGHT regional oximeter (CAS Medical Systems, Inc.) to collect rSO_2_. This regional oximeter monitors intracerebral rSO_2_ using NIRS and reflectance oximetry, displaying absolute rSO_2_ values and trends. Absolute oximetry values will be defined as rSO_2_ measured with an oximetry probe calibrated with a fixed arterial-to-venous blood ratio. In our study, only absolute oximetry data will be extracted and analyzed. We will install NIRS sensors on each side of the patient's forehead and measure rSO_2_ in both brain hemispheres. Baseline rSO_2_ will be recorded before the start of any medication and before induction of anesthesia, and subsequent rSO_2_ measurements will be recorded every 10 s until the last surgical suture is complete. At the end of the procedure, the data for the entire test cycle will be read via a memory stick through the USB port on the instrument. Moreover, the data collected (within the first two hours of surgery) will finally be presented as a line graph with data visualization software (GraphPad Prism) to visualize the temporal trend of changes in rSO_2_ in both hemispheres. Although we will perform dynamic acquisition of rSO_2_ values throughout the procedure for each patient, the visualization will be limited to 2 h after the start of the procedure due to the substantial variation in the time of the procedure between patients. The absolute change in percent change in rSO_2_ will be calculated by subtracting the baseline rSO_2_ value (T_0_) from the value measured at the end of the procedure (T_4_), multiplied by 100%. The investigators will collect rSO_2_ data from the sensors placed on the patient's left and right forehead.

### Measurement of PNDs

In 2018, a multidisciplinary expert working group on perioperative cognitive nomenclature standardized the terminology for cognitive changes associated with anesthesia and surgery [59], recommending the term "*perioperative neurocognitive disorders (PNDs)"* to describe patients with perioperative cognitive changes. The acronym PNDs is more relevant and reasonable based on the multidisciplinary consensus in neurology and the specialty of anesthesia on neurocognitive disorders. Further distinctions are made according to the point of onset and severity of cognitive impairment: (1) *Preexisting NCD*: a measurable, objective cognitive impairment that exists preoperatively; (2) *Postoperative delirium (POD)*: occurring within one week of surgery or before discharge and meeting the Diagnostic and Statistical Manual of Mental Disorders-5 (DSM-5) criteria for delirium; (3) *Delayed neurocognitive recovery (DNR)*: a cognitive impairment that exists within 30 days of surgery; (4) *Postoperative NCD*: cognitive impairment present from 30 days to 12 months postoperatively. It was also divided into *postoperative mild NCD* and *major postoperative NCD* according to the degree of cognitive impairment. (5) Cognitive impairment first diagnosed more than 12 months after surgery is referred to as *mild NCD* and *severe NCD*, depending on its severity, without the qualifier "postoperative" because it is difficult to determine whether the cognitive impairment occurring at this time is causally related to the previous anesthesia and surgery. The combination of delayed neurocognitive recovery, the cognitive decline within 30 days of surgery, and cognitive decline between 30 days and 12 months after surgery correspond to our traditional definition of "*postoperative cognitive dysfunction (POCD)*." In this study, the MMSE will be used to measure the occurrence of DNR in patients 1 d before and 1, 3, and 7 d after surgery, respectively (Table 2).

**Table 2:** Postoperative cognitive outcome and perioperative brain injury marker testing

**Variables**	MMSE	DNR	NSE	IL-6
Preoperative 1 d	**√**	**√**	**√**	**√**
Postoperative 1 d	**√**	**√**	**√**	**√**
Postoperative 3 d	**√**	**√**	**/**	**/**
Postoperative 7 d	**√**	**√**	**/**	**/**

*DNR* Delayed Neurocognitive Recovery, *MMSE* Mini-mental State Examination, *NSE* Neuron Specific Enolase, *IL-6* Interleukin-6

The MMSE is one of the most influential and popular international screening instruments for cognitive impairment, covering time, place orientation, immediate memory, attention, computation, short-term memory, language, and visuospatial structure skills. However, a capping and learning effect exists for the MMSE. The MMSE is insensitive to subtle changes in cognitive function that may occur after surgery. Thus, the MMSE is more suitable for preoperative screening in PND studies. The MoCA covers a broader range of cognitive domains than the MMSE, which makes it seems to be more accurate and sensitive than the MMSE for the determination of mild cognitive impairment. But our previous pilot study showed that the MoCA is too difficult and time-consuming to implement. Therefore, we will choose the MMSE as the assessment tool for postoperative cognitive function outcome (The patients with postoperative MMSE score ≤ 23 and a decrease of more than 2 points from the baseline value were defined as DNR). In addition, as a secondary outcome measure, we can accept the limitations of the MMSE.

### Measurement of PaCO_2_ and arterial blood gas analysis

Immediately after tracheal intubation, the minute ventilation will be adjusted to achieve PETCO_2_ concentrations of 45–55 mmHg in Group H and 35–40 mmHg in Group N. Due to the presence of alveolar dead space, PETCO_2_ maybe 5 mmHg-10 mmHg lower than PaCO_2_, and thus we will measure PaCO_2_ by performing an arterial blood gas (ABG) analysis and further adjust ventilation to achieve the desired PaCO_2_ target range. In addition, we will set 7.2 as the lowest pH limit for Group H. PaCO_2_ and the PETCO_2_ gradient will be maintained at constant levels as much as possible throughout the procedure. If the concentration of PaCO_2_ must be reassessed, for example, ventilation settings must be adjusted, additional ABG samples will be collected at the discretion of the anesthetist. Finally, at the end of the procedure, ABG samples will be collected to record PaCO2 values accurately and to assess whether PaCO_2_ is maintained within target values.

The Roche Cobas b 123 blood gas analyzer measures pH, PaCO_2_, Na^+^, K^+^, Cl^−^, Ca^+^, erythrocyte pressure (Hct), and metabolites (Glu and Lac) in whole blood using a selective motor technique. The pH, PaCO_2_, Na^+^, K^+^, Cl^−^, and Ca^+^ will be measured using the potentiometric method, erythrocyte pressure will be measured using the conductivity method, and PaO_2_ and metabolites will be measured using the current method. The built-in blood gas analyzer module measures total hemoglobin (tHb), hemoglobin derivatives (O_2_Hb, HHb, COHb, and MetHb), oxygen saturation (SO_2_), and bilirubin (Bili) levels using spectrophotometric methods. Notably, sodium heparin is the only anticoagulant allowed for use in the analysis with the Cobas b 123 system. Other anticoagulants, such as citrate, oxalate, fluoride, and ammonia-containing anticoagulants, exert significant effects on blood pH and other parameters and, therefore, should not be used. In addition, blood used for the analysis must be collected from the blood vessels of eligible persons. Under no circumstances should pressure be applied to the puncture site because the blood will begin to clot prematurely if the blood sample is mixed with tissue fluid, even if the sample collection vessel is adequately heparinized. The specimen should be sent for analysis immediately after collection and, in principle, should be tested within 15 min.

### Collection and analysis of venous blood samples

Venous blood samples are collected for two primary purposes: one is for blood gas analysis, and in collaboration with the ABG analysis at four-time points from T_1_ to T_4_, the CERO_2,_ and Da-jvO_2_ at the four-time points will be calculated using the Fick formula. The second objective is to determine brain injury marker levels to explore whether the interventions affect them and their association with postoperative cognitive function outcomes.

*Fick formula*:

$$CaO2=Hb\times 1.36\times SaO2+0.0031\times PaO2$$

$$CjvO2=Hb\times 1.36\times SjvO2+0.0031\times PjvO2$$

$$Da-jvO2= CaO2-CjvO2$$

$$CERO2:\left(CaO2-CjvO2\right)\div CaO2\times 100\%$$

*(CaO*_*2*_ *, SaO*_*2*_ *, and PaO*_*2*_ *are arterial oxygen content, arterial oxygen saturation, and arterial oxygen partial pressure, respectively; CjvO*_*2*_ *, SjvO*_*2*_ *, and PjvO*_*2*_ *are internal jugular venous oxygen content, internal jugular venous oxygen saturation, and internal jugular venous oxygen partial pressure, respectively; Hb represents hemoglobin concentration.)*

Venous blood samples will be collected from the bulb of the jugular vein preoperatively (after induction of anesthesia and before the start of surgery) and 24 h postoperatively, and the serum IL-6 and NSE concentrations will be measured using ELISAs. After the induction of anesthesia, a puncture will be made through the middle of the right internal jugular vein in the direction of the distal end, and a tube will be placed under ultrasound guidance in the reverse direction up to the bulb of the jugular vein for blood sample collection. The resistance encountered, and the depth of 15 cm will be used as the markers for entering the jugular vein bulb. Before and after the operation, 4 mL of blood samples will be collected from the bulb of the jugular vein and then centrifuged at 4000 r/min for 10 min at 4 °C after 30 min of incubation.

### NSE

NSE in the cytoplasm of neurons and neuroendocrine cells has the primary function of participating in glycolysis and catalyzing the production of phosphoenolpyruvate. Under normal conditions, the level of NSE in body fluids is shallow. However, following nerve cell injury, a large amount of NSE leaks from the intracellular space to the intercellular space and enters the cerebrospinal fluid and blood circulation. Therefore, an increase in the NSE level in body fluids indicates nerve cell damage and can be used as an essential predictor to determine the extent of neuronal damage [60]. NSE is a marker of neurons and neuroendocrine cells, with levels ranging from high to low in the brain, spinal cord, and peripheral ganglia. NSE accounts for 1.5% of all soluble proteins in the brain and is present at much lower levels in peripheral nerves than in brain tissue. Hans et al. [61] confirmed that NSE release was significantly and positively correlated with the number of dead neuronal cells but not with glial cells. Thus, in individuals with acute cerebrovascular diseases, such as cerebral hypoxia–ischemia, cerebral hemorrhage, and cerebral edema, the main pathological changes resulting from the injury are necrosis of neurons and disintegration of neurospinal phospholipid, and the NSE in the cytoplasm of necrotic neurons will be released directly into the cerebrospinal fluid (CSF), which increases the concentration of NSE in the CSF and subsequently leaks into the plasma through the damaged blood-cerebrospinal fluid barrier, all of which have been confirmed by animal experiments [62]. In addition, a comparison of blood and CSF specimens showed that serum NSE measurements replace CSF measurements and that the serum NSE level is a sensitive indicator of brain parenchymal damage [63]. Notably, Rasmussen et al. measured NSE levels in perioperative serum samples and assessed preoperative and postoperative cognitive function in 15 patients undergoing coronary artery bypass grafting, which concluded that an increase in serum NSE levels were strongly associated with early postoperative cognitive dysfunction [64].

### IL-6

Central nervous system inflammation significantly contributes to postoperative cognitive dysfunction, and increased expression of inflammatory factors often suggests early cognitive impairment [39]. IL-6 is a cytokine with a wide range of biological activities and is essentially a glycoprotein consisting of 184 amino acids that play an essential role in the inflammatory response of the body. The cells that synthesize IL-6 in the brain are astrocytes, microglia, and endothelial cells, and the cells that synthesize IL-6 in the periphery are mononuclear macrophages and lymphocytes [14]. Serum IL-6 levels are also correspondingly elevated after brain injury, which may be related to the increased early or ultra-early synthesis of IL-6 in the brain, disruption of the blood–brain barrier, and increased intestinal permeability to bacterial toxins. Weaver et al. [15] performed a rigorous regression analysis and found that elevated plasma levels of IL-6 are closely related to cognitive impairment and are a risk factor for cognitive impairment. Using transgenic mice overexpressing IL-6, the authors found that IL-6 plays a causal role in neuropathophysiological aspects such as reduced memory and learning abilities. This result further confirms that detecting IL-6 levels in plasma contributes to the early detection of cognitive impairment.

In summary, changes in the levels of both IL-6 and NSE may be used as markers of brain injury, and NSE has high sensitivity and specificity. In addition, recent evidence from animal experiments and clinical studies also supports the possibility of a close association between the levels of these two proteins and early postoperative cognitive dysfunction, which can be used as early warning indicators of postoperative cognitive dysfunction. Therefore, these two markers will be selected in this study to assist in assessing the extent of brain injury and to explore the potential cerebral protective role of PHC and the possible mechanisms by which PHC improves early postoperative cognitive function outcomes.

### Standardized perioperative care

#### Anesthesia induction

Through the standardized preoperative visit and corresponding optimal preoperative preparation, the patient will meet the requirements for elective surgery and fasting time, will not be administered any anticholinergic drugs or anti-5-hydroxytryptamines for 30 min before surgery, and will then be transported to the operating room. After admission, invasive arterial blood pressure (IABP), electrocardiogram (ECG), heart rate (HR), partial pressure of end-expiratory carbon dioxide (PETCO_2_), body temperature (T), pulse oximetry (SpO_2_), and electroencephalographic bispectral index (BIS) will be routinely monitored with a multifunctional anesthesia monitor connected to a brain tissue oxygen saturation (rSO_2_) monitor, and peripheral venous access will be opened. Atropine at 0.3–0.5 mg, sufentanil at 0.2–0.4 μg/kg, propofol at 1.5–2.0 mg/kg will be injected intravenously, and rocuronium at 0.6–0.9 mg/kg will be injected intravenously when the BIS value decreases to 45–55. After reaching the inotropic time, tracheal intubation will be performed, and mechanical ventilation will be connected to the anesthesia machine after confirming that it is placed in the trachea. After anesthesia, a retrograde puncture tube will be placed through the right internal jugular vein to the bulb of the internal jugular vein, and the tube will be sealed with heparin for blood sampling (the rate of blood collection from the bulb of the jugular vein is < 2 mL/min). A radial artery puncture tube will be placed to collect arterial blood samples.

#### Anesthesia maintenance

The MAC value was maintained between 1.1–1.3 by inhalation of 1.5%-2.0% sevoflurane and 60% N_2_O, the BIS value was held between 40–60, and remifentanil was continuously pumped at a starting rate of 0.01 µg/kg/ min. The pump speed of remifentanil was adjusted according to the fluctuation of intraoperative blood pressure (the maximum dose was not more than 1ug/kg/min), and the anesthesiologist added sufentanil at a quantity of 5 µg each time according to the fluctuation of the patient's hemodynamics. The inhalation of sevoflurane, N_2_O, and remifentanil will be stopped when the skin is sutured. After the patient's consciousness was clear, muscle strength and throat reflex recovered, the endotracheal tube was removed by adequate sputum suction, and the patient was sent to the Post Anesthesia Care Unit (PACU). The use of any awaking drug was prohibited if it was not necessary during the recovery period after surgery. Notably, the intraoperative depth of anesthesia should be maintained as stable as possible because the relationship between the depth of anesthesia and postoperative cognitive function outcome is highly controversial. The results of previous clinical studies and meta-analyses are contradictory, with some studies suggesting that a shallower depth of anesthesia is more favorable for improved postoperative cognitive function outcomes [65, 66], some confirming that a deeper level of anesthesia is associated with better cognitive function, especially the ability to process information, 4–6 weeks after surgery [67], and some findings even showing no significant correlation between depth of anesthesia and postoperative cognitive function [68]. Therefore, we should exclude the effect of the anesthesia depth on the study as much as possible. After tracheal intubation, the respiratory rate and tidal volume will be adjusted, and PaCO_2_ will be maintained within the respective target values in both groups.

#### PACU and ward

After the operation, the patient will be returned to the PACU for observation of awakening. After transfer to the PACU, the anesthesia nurse will connect the patient-controlled intravenous analgesia (PCIA) device and the drug formulation as follows: 2 µg/kg sufentanil and 0.3 mg of ramosetron, diluted to 100 ml using saline). Parameters are set as follows: a single compression dose of 2 ml/time, lock time of 15 min, and maximum dose of 8 ml per hour. The anesthesia nurse will assess the patient's pain score using the NRS method. When the patient's NRS score is ≥ 4 at rest, the PCIA analgesic device is pressed, and if the NRS score is ≥ 4 after the first press for 10 min, 5 µg of sufentanil will be administered intravenously as remedial analgesia until the pain score is < 4. When the patient is fully conscious, and the limbs can perform intended activities, the patient will be given air inhalation for observation for 1–2 min, and if the oxygen saturation is maintained above 95%, the patient will be transferred out of the PACU. On a postoperative day, nurses will assess pain scores in the surgical ward using the same NRS for all patients. All patients will receive 50 mg of flurbiprofen ester administered at 24 h intervals. The ward nurses are unaware of the grouping of patients and their interventions. Preoperative admission instruction will be put into practice: patients and family members assess pain scores themselves postoperatively, and again, the PCIA analgesia device is pressed when the NRS score is ≥ 4. If the NRS score is ≥ 4 after the first press for 10 min, 5 µg of sufentanil will be administered intravenously as remedial analgesia until the pain score is < 4.

### Sample size calculations

According to data previously reported by our institution, rSO_2_ values at wakefulness range from 60 to 80% for elderly patients with NFBF, consistent with previous studies [69]. Although the earlier studies of cerebral oxygen monitoring did not address the point of "NFBF," we obtained the same rSO_2_ interval in the preoperative awake state, which does not mean that this discussion is meaningless. However, the natural state of elderly patients with NFBF may not be significantly different from the average population due to the body's compensation, but when entering the general anesthesia and surgery state, the lack of cerebrovascular self-regulatory reserve capacity and the regional cerebral microcirculatory disorders of these patients only become apparent with the intensification of the systemic stress response and the increase of hemodynamic fluctuations.

Interestingly, a "15%" change in rSO_2_ values at the end of surgery relative to baseline values in both groups is considered clinically significant [70]. However, we need to state that "15%" is a research inference made for the anti-Trendelenburg position, and the laparoscopic colorectal surgery in this study was the Trendelenburg position. Due to the apparent position difference, we cannot ignore the secondary changes in cerebral perfusion and CBF. Continuing to choose the "15%" cutoff may overestimate the effect on the intervention group. Therefore, we used the more conservative "10%" relative change as the clinically significant margin in our trial. A baseline rSO_2_ of 60% to 80% was used, and the absolute change in rSO_2_ from baseline to the end of surgery was assumed to be 0% in the control group according to the pilot study and 8% in the intervention group (a 10% increase from baseline rSO_2_ of 80%). The sample-size calculation for the test of difference was performed with the use of PASS 15.0.5 software, and the total sample size needed for an unpaired t-test was calculated to be 68 at a two-sided significance level of 5% and 90% power. Considering the loss of a follow-up rate of 10%, our final sample size will be 76 (evenly distributed between the two groups).

### Statistical analysis

Data will be analyzed using Statistical Package for Social Science 25.0 software. The Shapiro–Wilk test is used to test the normality of continuous variables, expressed as means and SDs if they are normally distributed and as medians and interquartile ranges if they are non-normally distributed. Baseline, Intraoperative, and postoperative indicators between the intervention and control groups are analyzed by t-tests, Mann–Whitney U test, chi-square test, continuous corrected chi-square test, or Fisher's exact test (Table 3). All primary and secondary outcomes analysis will be based on intention-to-treat (ITT) which means all patients who accept randomization will be included in the final statistical analysis, regardless of whether they received the intended intervention. We will also conduct per-protocol (PP) analysis for primary and secondary outcomes. ITT analysis usually attenuates differences between two groups, and PP analysis exaggerates differences between two groups. We will explore the consistency of ITT and PP analysis results and verify the results' reliability if they are consistent in the end. For the primary outcome, linear regression models will be used to generate unadjusted effect sizes with 95% confidence intervals. Preoperative baseline and intraoperative data are also included in the linear model to generate adjusted effect sizes and 95% confidence intervals. The analysis of repeated measures (rSO_2_, Da-jvO_2_, CERO_2_) will be performed using generalized estimating equations (GEE), with patient code (ID) as the within-subject variable, time as the between-subject variable, and rSO_2_, Da-jvO_2_, and CERO_2_ as the dependent variable. The ACE-R scores of the intervention and control groups will also be analyzed by GEE. What we need to be clear is that we will not adjust for multiple comparisons of secondary outcomes at numerous measurement time points, and we will focus on the separate effects of intervention because these results are exploratory. The incidence of CDE and DNR between the intervention and control group will be analyzed by the chi-square test, continuous corrected chi-square test, or Fisher's exact test. The serum NSE and IL-6 concentrations between the intervention and control groups are analyzed by covariance (ANCOVA) analysis, and the least-squares mean (LSM) difference and 95% CI will be estimated. Postoperative adverse events will be analyzed using the safety analysis set, which refers to patients who received at least one intervention in their group. Postoperative adverse events are categorical data, and the chi-square test, continuous corrected chi-square test, or Fisher's exact test will be used for statistical analysis.

**Table 3:** Baseline and intraoperative characteristics to be compared between the two groups

Baseline Characteristics	Intraoperative characteristics
**Age** (year)	**Anesthesia time** (min)
**Sex**	**Operation time** (min)
• Male	**Pneumoperitoneum time** (min)
• Female	**Surgical method**
**Weight** (kg)	**Intraoperative hypotensive events**
**BMI** (kg/m^2^)	**Bradycardia events**
**ASA** physical status	**Blood transfusion events**
• I	**Blood loss** (ml)
• II	**Infusion quantity** (ml)
• III	**Vasoactive drug use** (ug)
**rSO**_**2**_ (%)	• Ephedrine
**PaO**_**2**_ (mmHg)	• Phenylephrine
**PaCO**_**2**_ (mmHg)	• Norepinephrine
**Hb** (g/L)	• Epinephrine
**Ejection fraction** (%)	**Opioid consumption** (ug)
**Stroke volume** (ml)	• Remifentanil
**Comorbidity**	• Sufentanil
• Hypertension (I/II/III)	• Tramadol
• Diabetes	• Oxycodone
• COPD	**Blood gas analysis of different period (T**_**1**_**/T**_**2**_**/T**_**3**_**/T**_**4**_**)**

Blood gas analysis: pH/ PaCO_2_/ PaO_2_/ Hb/ Lac/ Glu

## Anticipated results

The expected outcomes of this study are divided by objective. First, in terms of rSO_2_ and cerebral oxygen metabolism, when examining the primary outcome of an absolute change in the percent change in rSO_2_ from baseline to the completion of surgery between intervention and control groups, significant fluctuations in rSO_2_ may not be observed in the control group throughout the surgery, but under the dual stress conditions of general anesthesia and surgical trauma, reasonable speculation is that older patients with fragile brain function most likely have a trend of a decrease during surgery, which may be attributed to the poorer cerebral vascular self-regulation and lower functional reserve in this subject population, therefore, that the absolute difference between their postoperative rSO_2_ values and their preoperative baseline values may be < 0. However, in the intervention group, PHC mediated by small tidal volume ventilation may exert a comprehensive cerebral protective effect through potential mechanisms such as cerebral vasodilation, improvement of cerebral microcirculation, maintenance of the balance of cerebral oxygen supply and demand, mitigation of free radical-induced damage, and inhibition of the inflammatory response and apoptosis. These changes may result in an increasing trend in the intraoperative changes in rSO_2_ in the intervention group. The absolute change of rSO_2_ values in the control and intervention group are significantly different, which is supported by evidence in a previous related study [71]. As mentioned previously, these possible mechanisms have been clarified in animal studies and clinical trials in recent years, and the selection of older patients with NFBF as subjects in the present trial to investigate the association between PHC and rSO_2_ is a reasonable approach and extension based on the existing research evidence.

In addition, we will also perform statistical analyses (calculated using Fick's formula) of the relevant indicators of cerebral oxygen metabolism, "DajvO_2_ and CERO_2_" because the exploration of the potential correlation mechanism *"PHC → improves cerebral oxygenation → improves postoperative cognition"* is another critical objective of this study, and the significant correlation between cerebral oxygen metabolism and postoperative cognitive dysfunction has been confirmed [72, 73]. Although few previous studies related to PHC and cerebral oxygen metabolism have been conducted, studies on the relationship between the two have produced contradictory results, with evidence of positive, negative, and no correlations having been reported [12, 74–76]. However, we maintain that the final findings will support the hypothesis that PHC accompanied by a higher rSO_2_ improves cerebral oxygen metabolism and reduces the incidence of DNR, thus improving cognitive function outcomes. In addition, our analysis of serum NSE and IL-6 levels by collecting blood samples may reveal significant differences between the two groups. The intervention group may be able to mitigate perioperative brain injury through the multifaceted brain-protective mechanism of PHC, and NSE and IL-6 levels will be lower than those in the control group as predictors of brain injury.

According to the logical stratification of this study, we included postoperative cognitive outcome as a secondary outcome, and graded these parameters quantitatively based on MMSE scale scores to assess the postoperative cognitive function status of the two groups and combined with the differences in the effects of PHC interventions on "rSO_2_, DajvO_2_, CERO_2_, NSE, and IL-6 levels" in the two groups, a multifaceted and multilevel approach will be used to verify the proposed mechanism of *"PHC → improves cerebral oxygenation → improves postoperative cognition."*

Other secondary findings, including surgical prognosis, postoperative complications, the incidence of adverse effects, and length of hospital stay, lack relevant clinical evidence and theoretical support and require us to explore the intrinsic associations through the specific trial process.

## Discussion

We will conduct a prospective, single-center, single-blind RCT to assess the effects of interventions on rSO_2_ in patients undergoing laparoscopic colorectal cancer surgery. We will use the absolute change between postoperative and preoperative rSO_2_ values as the primary outcome of the study. Four-time points, T_1_, T_2_, T_3_, and T_4_, are selected to record real-time rSO_2_ and to perform the blood gas analysis and calculations of metabolism-related indicators of cerebral oxygenation. We plan to promptly adjust intraoperative PaCO_2_ and pH on the blood gas results. Notably, the previous clinical focus of hypercapnia has always been on the acidosis and subsequent hyperkalemia that it causes, but one study reported no significant clinical association between hypercapnia and hyperkalemia [77]. In addition, we will dynamically monitor the changes in blood gas parameters at four intraoperative points to ensure trial safety and compliance with ethical guidelines.

On the other hand, the observation of indicators related to cerebral oxygen metabolism and the examination of preoperative and postoperative levels of brain injury markers (NSE and IL-6) provides a further exploration of the comprehensive mechanism of action of PHC to improve the overall cerebral oxygenation function and exert cerebral protective effects. Notably, the majority of previous RCTs on brain protection and postoperative cognition have focused on older adults with good underlying brain function and have used "a history of cerebrovascular accident and/or cognitive dysfunction" as the main exclusion criteria, suggesting that the potential typical beneficiary group of the trial results could not be verified and limiting the externality of the clinical results and contradicting the pragmatic trials currently advocated in the world. Therefore, this study is undoubtedly a potent supplement.

In addition, during the first two hours of the procedure, we will visualize the trend of changes in cerebral oxygenation through the cerebral oxygen monitor to reflect further how the effect of PHC on rSO_2_ changes over time: whether the trend is increasing or decreasing or whether it shows irregular and dynamic fluctuations.

The relationship between PaCO_2_ and CBF has been well documented in recent years, both in terms of underlying physiological mechanisms and in clinical practice, but the relationship between PaCO_2_ and rSO_2_ has not been clarified. Many factors affect rSO_2_, such as cardiac output, volume per beat, MAP, Hb affinity for oxygen, cerebral vascular self-regulation, and surgical position adjustment. Among them, PaCO_2_ may exert a direct and significant effect on the affinity of hemoglobin for oxygen by regulating pH and subsequently through the Bohr effect. Throughout the procedure, we can control for potential confounders such as PaCO_2_, pH, MAP, and intraoperative body position. We will make preoperative baseline adjustments to the subject's MAP, Hb level, and cardiac output. However, intraoperative changes in cardiac output, the brain's self-regulatory effect on the arteriovenous blood volume ratio, the effect of laparoscopic surgery on thoracic pressure, and the subsequent effects on cardiac output, volume per beat and systemic vascular resistance cannot be measured objectively and realistically.

In conclusion, although an increasing number of studies have explored the relationship between PaCO_2_/PHC and rSO_2_ in recent years, the current research evidence only provides an objective description of the clinical phenomenon, as shown in the Table 4. The specific underlying mechanism of action remains unclear, and we should not draw hasty conclusions.

**Table 4:** Summary of related studies on the relationship between mild hypercapnia and rSO_2_

**Author**	**Year**	**Subjects**	**Trial**	**Outcomes** (Mild hypercapnia)
Eastwood et al. [78]	2016	Cardiac arrest	Prospective double crossover physiological study	Increases cerebral oxygenation
Akca et al. [79]	2002	Abdominal or orthopaedic surgery	RCT	Improves tissue oxygenation; Increase cerebral oxygen saturation; Reduce the incidence of wound infection
Murphy et al. [27]	2014	Shoulder surgery	RCT	Higher intraoperative rSO_2;_ Lower incidence of CDE
Park et al. [80]	2021	Gynecological Laparoscopic surgery	RCT	Increases rSO_2_
Wong et al. [71]	2020	Elective major surgery	RCT	Associated with a stable increase in rSO_2_

**Fig. 3:**
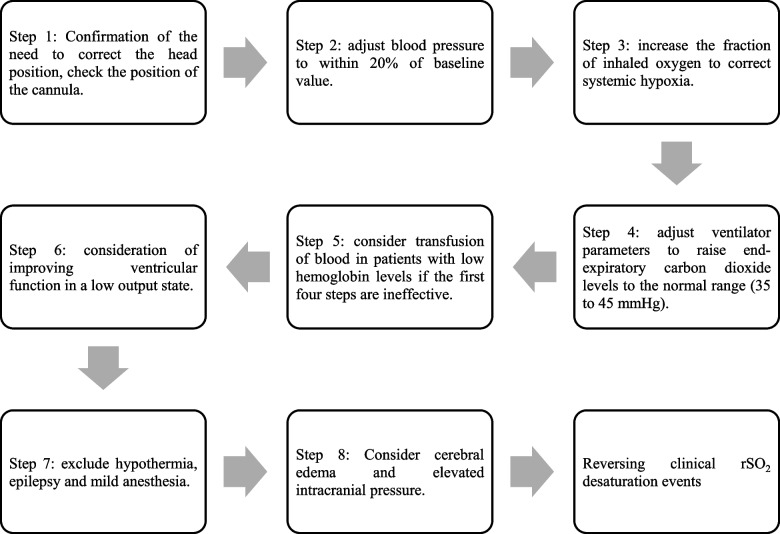
Flowchart of the algorithm for the eight-step cerebral oxygen intervention

Notably, although theoretical absolute and relative saturation thresholds for the need for rapid intervention have been previously proposed [35], these thresholds have not been validated, and a consensus regarding the indication and timing of interventions is lacking. In our study, we are not yet able to predict the trend and the extent of changes in rSO_2_ in the intervention and control groups, and we will not have intervened in intraoperative rSO_2_ changes if the control group exhibits a slight reduction in rSO_2_ compared to the baseline value and the attending anesthesiologist does not have a clear rSO_2_ target. However, we will consider CDE (≤ 55% absolute change, or a ≥ 20% decrease relative to the baseline) as a marker for administering clinical interventions and stop the intervention when the rSO_2_ increases above the threshold. Although no precise timing for the intervention and a standard intervention has not been established, we will adopt an "*eight-step approach*" for the cerebral oxygen intervention, as shown in the Fig. 3, based on evidence from prior mainstream studies, which has been confirmed to be an effective interventional process in reversing rSO_2_ desaturations [35]. Importantly, for patients with CDE, we will discontinue the trial for emergency unblinding for an intervention, but we will not directly exclude the data from the trial. We will perform an ITT statistical analysis and compare the results with the PP statistical analysis. If the results of both analyses are consistent, the trial will be considered more reliable. In addition, after patients have been randomized into groups, any scenario that might endanger patients' lives and substantially interfere with rSO_2_, such as a massive intraoperative hemorrhage (bleeding of 800 ml or more in a short period or 20% of the total circulating blood volume), severe subcutaneous emphysema, and shock (decompensated period), should promptly result in the discontinuation of the trial, and the ITT statistical analysis will also be needed.

The assessment of postoperative cognitive function, another important finding of this study, has important clinical implications for investigating the potential mechanism of " *PHC → improves cerebral oxygenation → improves postoperative cognition. "* In this trial, the occurrence of DNR is quantified by scoring using the MMSE scale. However, we need to be clear that if, in the final statistical analysis, we find that the cognitive status between the two groups is not statistically different, or if there is a statistical difference, there is a cognitive-related confounding factor such as ASA grade or age. This outcome is possible and acceptable because this trial is not originally designed to explore the quantitative relationship between hypercapnia and postoperative cognitive performance. Although previous clinical evidence for the association between intraoperative rSO_2_ and cognitive function supports the potential hypothesis of "*PHC → improves cerebral oxygenation → improves postoperative cognition*," as shown in Table 5, researchers have not determined whether the specific mechanism of action is a simple effect of rSO_2_ or a combination of rSO_2_, cerebral oxygen metabolism, brain injury, neuronal apoptosis, and neurotransmitter regulation. In conclusion, both basic studies and large-scale risk factor analyses controlling for other possible confounders of postoperative cognitive function are inadequate, and the specific mechanism of the effect of cerebral oxygen saturation on postoperative cognitive performance is likewise not supported by sufficient evidence.

**Table 5:** Summary of studies related to the association between rSO_2_ and postoperative cognitive function

Author	Year	Subjects	N	Oximeter	Outcomes
Trafidlo et al. [81]	2015	Lumbar spondylosis	43	INVOS 5100	NIRS cerebral oximetry may be helpful in reducing postoperative cognitive complications in patients operated on in the prone position
Murniece et al. [82]	2019	Spinal neurosurgery	34	INVOS 4100	The NIRS-based clinical algorithm may help avoid POCD in patients after spinal surgery
Casati et al. [83]	2005	Major abdominal surgery	122	INVOS 4100	A higher rSO_2_ improved MMSE scores in elderly patients undergoing major abdominal surgery
Slater et al. [84]	2009	Coronary artery bypass grafting	240	INVOS 5100B™	Intraoperative cerebral oxygen desaturation is significantly associated with an increased risk of cognitive decline
Colak et al. [85]	2015	Coronary artery bypass surgery	200	INVOS 5100c	Intraoperative cerebral oxygen saturation monitoring is beneficial for postoperative cognitive function outcomes, and rSO_2_ desaturation predicts POCD

In the future, based on the general direction of this study, we must strengthen the basic research on the effect of PHC on rSO_2_, clarify the specific mechanism, and further promote the popularization of the clinical application of NIRS. Basic research should be combined with evidence from multicenter, large-sample clinical trials to prove the strong association between PHC and rSO_2_. On the other hand, we should extend the hypothesis of "PHC-rSO_2_↑" to the postoperative cognitive function of patients and explore whether rSO_2_ is a beneficial factor for postoperative cognitive performance by performing RCTs or observational cohort studies with larger sample sizes.

Moreover, much more research is needed to explore the effects of PHC on cerebral oxygenation and postoperative cognitive performance, and several questions remain to be answered, as described below.①As a highly metabolic and oxygen-consuming organ, the brain has its own complex hemodynamic regulatory mechanisms, and the effect of PHC on it will likely vary substantially under different pathophysiological conditions. The pathophysiological states that might best benefit and the suitable and contraindicated populations must be determined according to the mechanism of action of PHC, which is the ultimate significance of basic research on PHC and the prerequisite for its clinical application.②After clarifying the beneficiary population, we must further develop a unified, standard processing specification, which we call the "*When/How (velocity* + *extent)/Duration*" algorithm: When should PHC be started? How can PHC standards be achieved? (in what way, at what speed, and to what extent) How long should PHC be maintained?③Having identified the applicable population and established the treatment criteria; we must further clarify the clinical safety and efficacy bounds of PHC. Based on the evidence from previous studies, the skylight effect of PHC on animal tests has been validated, and a reasonable range has been initially tested [6, 86], but all these parameters remain unclear in the clinical setting.

Only when the abovementioned issues are fully validated and solved can we genuinely apply PHC in the clinical setting and develop individualized protocols for specific patients, thus improving the significance of clinical practice and building a bridge between basic research and clinical application. In summary, the aging of the global population is gradually increasing, indicating that the number of older individuals requiring clinical and surgical treatment has increased simultaneously [87]. Moreover, this situation poses a significant challenge to healthcare institutions due to the unique characteristics of older individuals, such as declining physical functions, reduced reserve capacity of various tissues and organs, and degenerative changes in the central nervous system. Most previous studies on brain protection measures and related mechanisms are focused on basic research, while in clinical practice, they often explore measures in response to existing brain damage. Based on the predictability of NIRS technology, the current study monitors cerebral oxygenation to explore potential brain protection strategies, which have profound significance to the current world medical environment.

## Data Availability

Not applicable.

## Abbreviations

rSO_2_: Regional cerebral oxygen saturation
NIRS: Near-infrared spectrum
PHC: Permissive hypercapnia
NFBF: Nonacute fragile brain function
RCT: Randomized controlled trial
ASA: American Society of Anesthesiologists
BMI: Body mass index
TIA: Transient ischemic attack
AD: Alzheimer's disease
PD: Parkinson's disease
ARDS: Acute respiratory distress syndrome
PETCO_2_: End-tidal carbon dioxide tension
CDE: Cerebral oxygen desaturation events
MMSE: Mini-mental state examination
MoCA: Montreal Neurocognitive Assessment
CAM: Confusion Assessment Method
Da-jvO_2_: Difference in arteriovenous oxygen content
CERO_2_: Cerebral oxygen uptake rate
PNDs: Perioperative neurocognitive disorders
DNR: Delayed neurocognitive recovery
POD: Postoperative delirium
POCD: Postoperative cognitive dysfunction
ABG: Arterial blood gas
NSE: Neuron Specific Enolase
CSF: Cerebrospinal fluid
CBF: Cerebral blood flow
PACU: Postanesthesia care unit
PCIA: Patient-controlled intravenous analgesia
